# Accelerated Cobalt-Catalyzed *N*-Methylation via Microwave-Induced Rapid Formation of Active Species Using Methanol and Methanol-*d*_4_

**DOI:** 10.3390/molecules31071068

**Published:** 2026-03-24

**Authors:** Miki Takizawa, Takahiro Yamane, Akinobu Matsumoto, Takashi Miyazawa, Satoshi Horikoshi

**Affiliations:** 1 Department of Materials and Life Sciences, Faculty of Science and Technology, Sophia University, 7-1 Kioicho, Chiyoda-ku 102-8554, Tokyo, Japan; 2 Wisdom Pool Research Institute G.K., 32F Tokyo Midtown Yaesu Yaesu Central Tower, 2-1-1, Yaesu, Chuo-ku 104-0082, Tokyo, Japan

**Keywords:** microwave heating, electromagnetic wave effect, cobalt catalysis, *N*-methylation, *N*-trideuteromethylation, methanol, methanol-*d*_4_

## Abstract

The development of sustainable and environmentally benign *N*-methylation methodologies is essential for enhancing sustainable synthetic practice in pharmaceutical manufacturing. In this study, we demonstrate that microwave heating (MWH) markedly enhanced the efficiency of cobalt-catalyzed *N*-methylation using methanol or methanol-*d*_4_ as green C1 sources. Compared with conventional heating (CH), MWH enabled highly efficient syntheses of key pharmaceutical intermediates—including 6-dimethylamino-1-hexanol, imipramine hydrochloride, and butenafine hydrochloride—under milder conditions and shorter reaction times and without generating hazardous halogen-containing waste. UV–vis spectroscopic analysis revealed that MWH accelerated the transformation of Co(acac)_2_ into catalytically active Co species by approximately four-fold, providing a mechanistic basis for the enhanced reactivity. We hypothesized that this effect was caused by the selective microwave heating of the catalyst, which in turn promoted the rapid generation of catalytically active species. Notably, MWH also significantly improved the *N*-trideuteromethylation of amines using methanol-*d*_4_, achieving a 95% yield for imipramine-*d*_3_ hydrochloride versus 32% under CH. Molecular dynamics simulations indicated that methanol-*d*_4_ exhibited slower dipole relaxation and enhanced cluster fragmentation under microwave fields, improving catalyst–substrate contact, while kinetic isotope effects stabilized reactive intermediates. These synergistic effects account for the pronounced microwave promotion observed in deuterated systems. Overall, the combination of MWH and cobalt catalysis offers an energy-efficient, waste-minimizing, and environmentally benign strategy for the scalable synthesis of both methylated and deuterated amines.

## 1. Introduction

Over the past five years, 394 new active pharmaceutical ingredients (APIs) have been launched globally, primarily in Europe, the United States, and Japan [1], with the pharmaceutical market size increasing to $1.7 trillion in 2024 [2]. However, global drug shortages are worsening, attributed to factors such as the lack of safe and affordable manufacturing methods, difficulties in sourcing high-quality raw materials, and a surge in pharmaceutical demand [3]. Small-molecule drugs and large-molecule drugs account for most of the pharmaceutical market [4]. Small-molecule drugs are generally molecules with a molecular weight of 700 Da or less that possess pharmacological activity and have simple chemical structures. Conversely, macromolecular drugs are characterized by their extremely large size, ranging from several thousand to hundreds of thousands of Da, with complex three-dimensional structures. While both types face the risk of supply shortages, low-molecular-weight drugs are more prone to chronic shortages due to reasons such as manufacturing cessation or withdrawal stemming from low profit margins [5]. Therefore, developing simple and safe manufacturing methods is essential for low-molecular-weight drug production. The primary main skeletons of small-molecule drugs include *N*-alkylamines, geometric isomers, and aldehydes. Over half of the top 200 best-selling small-molecule drugs possess an *N*-alkylamine skeleton [6]. Therefore, highly efficient *N*-alkylamine synthesis methods could help alleviate the global drug shortage. Thus, we focused on the simplest *N*-alkylation reaction among *N*-alkylamine synthesis methods.

Traditionally, *N*-methylation has been achieved mainly via S_N_2 reactions with methyl iodide [7] or by reductive *N*-methylation using formaldehyde and formic acid (Eschweiler–Clarke reaction) [8,9], both of which are widely employed in industrial processes. However, these methods often suffer from overalkylation, leading to reduced yields of the desired products and complicating purification. Moreover, the use of highly toxic formaldehyde, the need for excess amine substrates, and the generation of hazardous halogen-containing byproducts result in difficult workup procedures and increased production costs. Therefore, the development of efficient *N*-methylation reactions using less toxic methylating agents is highly desirable. In recent years, transition metal-catalyzed *N*-methylation via the borrowing hydrogen methodology has attracted considerable attention. In this approach, methanol serves as a methylating agent, and a transition metal catalyst promotes the in situ dehydrogenation of methanol to formaldehyde. The resulting formaldehyde condenses with an amine to form a transient imine intermediate, which is subsequently hydrogenated by the catalyst to give the *N*-methylamine product. Since water is the only byproduct, this reaction is particularly promising as an environmentally benign *N*-methylation method, especially when methanol is sourced through sustainable processes. To date, various transition metal-catalyzed *N*-methylation methods using methanol have been reported, employing catalysts based on cobalt [10,11], ruthenium [12,13,14,15,16,17,18,19,20,21,22,23,24,25], iridium [26,27,28,29,30,31,32,33,34,35,36], palladium [37], rhenium [38], iron [39,40], and rhodium [41]. Generally, these catalytic systems require heating at temperatures above 140 °C and reaction times exceeding 24 h to achieve high yields. Moreover, the elevated vapor pressure of methanol necessitates high-pressure reaction conditions, which remains a significant challenge for practical applications.

Since the latter half of the 20th century, numerous reports have demonstrated that combining MWH with organic synthesis reactions can enhance reaction efficiency. For example, in the Suzuki–Miyaura coupling reaction, while CH for 7 min resulted in only 8% yield, MWH increased the yield to 56% in just 5 min [42]. This reaction employs palladium as a transition metal catalyst, suggesting that the unique effects of MWH on metal catalysts contributed significantly to the improved reaction efficiency. By leveraging these advantages, it is anticipated that the severe reaction conditions typically required—such as heating above 140 °C, high-pressure operation, and prolonged reaction times—can be replaced with milder conditions, including lower temperatures, ambient pressure, and shorter reaction times. Thus, we investigated the effect of MWH on metal-catalyzed *N*-methylation reactions using cobalt catalysts. We compared the product yields obtained under CH and MWH to assess whether *N*-methylation could proceed efficiently under milder MWH conditions. Furthermore, to evaluate the practical utility of this method, we combined the use of MWH and transition metal catalysis to the synthesis of pharmaceutically relevant compounds.

In addition, deuterium-labeled compounds have found wide applications in both academic research and industrial processes [43,44]. Among them, *N*-trideuteromethylated amines are of particular interest as promising drug candidates [45]. The kinetic isotope effect arising from the difference between C–H and C–D bonds can retard metabolic degradation in vivo, potentially prolonging the half-life of pharmaceutical agents [46,47]. Consequently, deuterium-labeled drugs can help suppress toxicity and undesirable side effects by reducing the required dosage, without compromising biological activity. Conventionally, *N*-trideuteromethylamines are synthesized by reacting amines with CD_3_I or (CD_3_)_2_SO_2_ in the presence of stoichiometric or excess amounts of base [48,49]. However, these methods suffer from several drawbacks, including the use of expensive, highly toxic, and carcinogenic reagents, poor selectivity, and the generation of large quantities of hazardous waste. In contrast, deuterated methanol (CD_3_OD) is more readily available and offers a practical route to access trideuteromethylated compounds. Although transition metal-catalyzed trideuteromethylation reactions using methanol-*d*_4_ have been explored, the low reactivity of methanol-*d*_4_ [50] often results in prolonged reaction times and reduced yields. In this study, we found that microwave irradiation selectively heats the cobalt catalyst, thereby promoting the rapid generation of catalytically active species and potentially overcoming the intrinsic low reactivity of methanol-*d*_4_. To address these challenges, we examined the effect of MWH on the transition metal-catalyzed *N*-trideuteromethylation of amines using methanol-*d*_4_, targeting the synthesis of imipramine-*d*_3_ as a model compound.

## 2. Results and Discussion

### 2.1. Efficacy of Microwaves in N-Methylation Reactions

**Figure 1:**
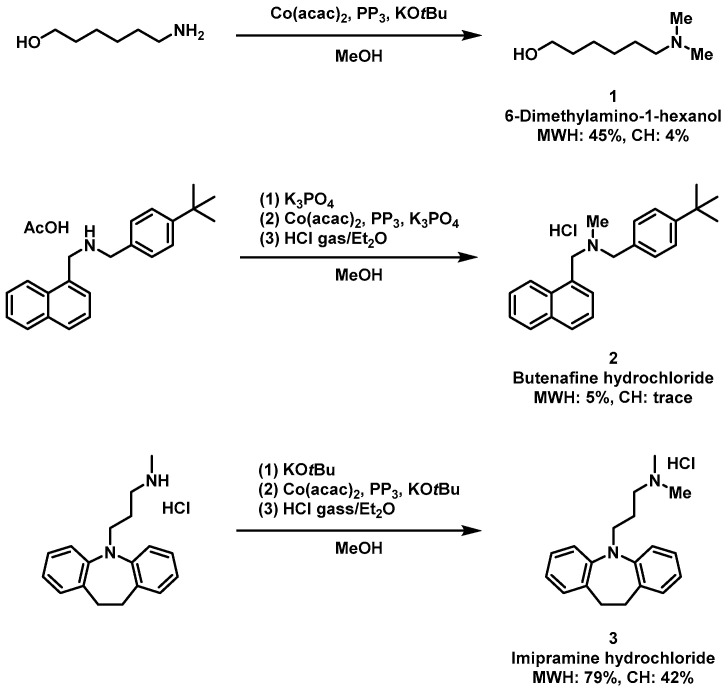
Comparison yields of 6-dimethylamino-1-hexanol, butenafine hydrochloride and imipramine hydrochloride synthesis by *N*-methylation reaction with Co(acac)_2_ catalysis using microwave heating (MWH) and conventional heating (CH).

Since there have been no reports on the application of microwave heating to Co-catalyzed *N*-methylation reactions, in this paper we investigated the usefulness of microwave heating by applying it to the previously reported *N*-methylation reaction using Co(acac)_2_ [10]. To demonstrate the applicability of this reaction in pharmaceutical processes, we selected 6-dimethylaminohexanol, a potential pharmaceutical synthetic intermediate, butenafine hydrochloride, an active ingredient in antifungal drugs, and imipramine hydrochloride, an active ingredient in antidepressants, as target compounds. 6-dimethylamino-1-hexanol, imipramine hydrochloride, and butenafine hydrochloride were investigated. The synthesis of 6-dimethylamino-1-hexanol was carried out at 100 °C for 4 h, yielding a 45% MWH and a 4% CH yield (Figure 1, entry 1). In other words, the use of MWH resulted in an 11.2-fold increase in yield. On the other hand, when butenafine hydrochloride was synthesized at 140 °C for 8.5 h, MWH was obtained in 5% yield, while CH synthesis barely progressed (Figure 1, entry 2). This result indicates that even when CH synthesis barely progresses, the reaction proceeds using MWH.

Furthermore, our investigation has not revealed any reports on the synthesis of butenafine hydrochloride using a homogeneous metal catalyst. The synthesis of butenafine hydrochloride is difficult due to the large steric hindrance around the nitrogen atom in the substrate. However, we predict that the high activation energy barrier caused by steric hindrance can be overcome with MWH, possibly by localized heating at the molecular level. When imipramine hydrochloride was synthesized at 100 °C for 1 h, MWH yielded 79% and CH yielded 42%. Using MWH increased the yield by 1.9-fold compared to CH.

**Figure 2:**
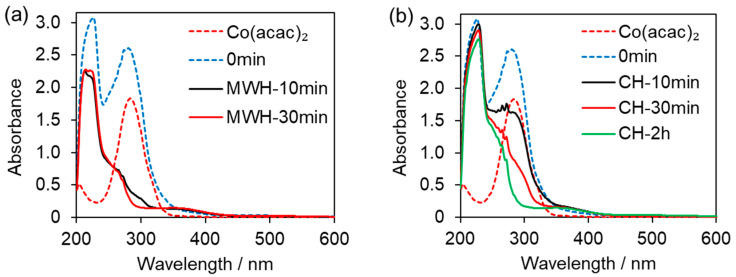
Comparison of UV-vis absorption spectra of Co(acac)_2_, PP_3_, and KOtBu in methanol solution before and after (**a**) MWH and (**b**) CH, and Co(acac)_2_ in methanol solution.

Next, we evaluated why MWH has a higher yield than CH in terms of the amount of catalytically active species in the transition metal catalyst. First, we measured the UV-vis absorption spectrum of a control (unheated) solution of Co(acac)_2_ dissolved in methanol. A peak with a maximum absorption at 284 nm was observed, which is believed to correspond to Co(acac)_2_ (Figure 2a). Next, we measured the UV-vis spectrum of a control (unheated) solution of methanol containing Co(acac)_2_, P(CH_2_CH_2_PPh_2_)_3_ (PP_3_), and KOtBu. The absorption intensity increased, but the wavelength remained the same. An additional absorption peak at 226 nm appeared, which is attributable to PP_3_ and KOtBu (Figure 2a). A methanol solution containing Co(acac)_2_, PP_3_, and KOtBu was then heated (80 °C) with MWH under a nitrogen atmosphere. After 10 min of microwave irradiation, the 284 nm peak attributable to Co(acac)_2_ was significantly attenuated, with a decay rate of over 90% (Figure 2a).

On the other hand, when a methanolic solution containing Co(acac)_2_, PP_3_, and KOtBu was heated with CH, the 284 nm peak attributable to Co(acac)_2_ was reduced, as in microwave heating, but the decay rate was only 37% (Figure 2b). After 30 min of heating, the peak intensity decayed by 71%, and after 2 h, it decayed by over 90%. Microwave heating can transform Co(acac)_2_ in a very short time, with 10 min of microwave heating being equivalent to 2 h of conventional heating. This indicates that microwave heating can quickly convert catalytic raw materials into catalytically active species.

We hypothesized that this phenomenon was due to selective heating of the catalyst by microwave irradiation. To test this hypothesis, we preheated a methanol solution containing Co(acac)_2_, PP_3_, and KO*t*Bu at 120 °C for 30 min using an aluminum block heater. 6-Amino-1-hexanol was then added, and the reaction temperature was set to 100 °C for 4 h. The desired product, 6-dimethylamino-1-hexanol, was obtained in 30% yield. This result suggests that by preforming the catalytically active species by raising the solution temperature to 120 °C, the activation efficiency from the catalyst precursor was significantly improved, although not as high as by microwave heating.

As shown in Figure 1, entry 1, when conventional heating was performed at 100 °C for 4 h without preheating, the yield was only 4%, suggesting that preheating at a high temperature accelerated the rate-determining step of catalyst formation and significantly improved the synthesis yield. Based on these results, it is highly likely that microwave heating selectively heated Co(acac)_2_, facilitating the rapid generation of catalytically active species. Furthermore, the promotion of adsorption and desorption (contact efficiency) by selective heating of the catalyst surface may also contribute to the yield improvement.

### 2.2. Efficacy of Microwaves in Deuteration Using N-Methylation Reactions

**Figure 3:**
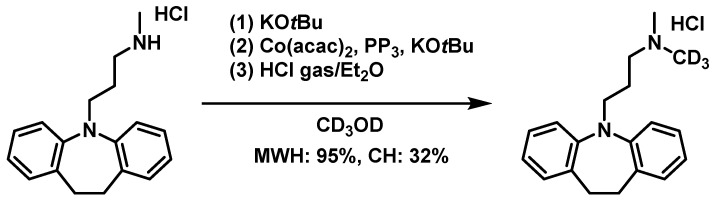
Comparison yields of *N*-methylation reaction with Co(acac)_2_ catalysis to synthesize imipramine-*d*_3_ hydrochloride by microwave heating (MWH) and conventional heating (CH).

All *N*-methylation reactions shown in Figure 1 demonstrated high yields using microwave irradiation. Imipramine hydrochloride is used as a reagent in antidepressants, and if deuterated methylation could be easily performed, it would reduce the number of antidepressant doses and associated side effects [46,47]. However, deuterated methylation has low reactivity and low productivity in actual pharmaceutical processes. For this reason, we hypothesized that the synthesis of imipramine-*d*_3_ hydrochloride using MWH could improve productivity, and comparative experiments were performed using MWH and CH (Figure 3).

Using CH, due to the higher bond dissociation energies of C–D and O–D bonds compared to C–H and O–H bonds, a longer reaction time was required than in the corresponding protium system. Under conditions of 100 °C for 6 h, the yield using MWH reached 95%, while that using CH was only 32%. Amazingly, MWH improved the reaction efficiency by approximately three-fold, and the microwave promotion effect was more pronounced than that observed in the synthesis of non-deuterated imipramine hydrochloride. These findings suggest that MWH can serve as a resource-efficient approach applicable to the industrial synthesis of deuterated compounds.

### 2.3. Mechanism of Microwave-Enhanced Effect in Deuteration

In the *N*-methylation reaction, the particularly high yields with methanol-*d*_4_ were attributed to differences in the microwave response behavior compared to methanol. An Agilent Technologies ENA vector network analyzer was used to measure the dielectric constant (*ε*′) and dielectric loss (*ε*″) for methanol and methanol-*d*_4_ at 25 and 60 °C and their respective tan *δ* values were calculated (Table 1). At 25 °C, the *ε*′ values for methanol and methanol-*d*_4_ were 23.3 and 21.4, respectively, indicating a higher microwave absorption rate for methanol. However, the tan *δ* values for methanol and methanol-*d*_4_ were 0.60 and 0.67, indicating a higher microwave heating efficiency for methanol-*d*_4_. Even at 60 °C, methanol exhibited higher microwave absorption than methanol-*d*_4_, but the tan *δ* value for methanol-*d*_4_ (0.39) was higher than that for methanol (0.36).

**Figure 4:**
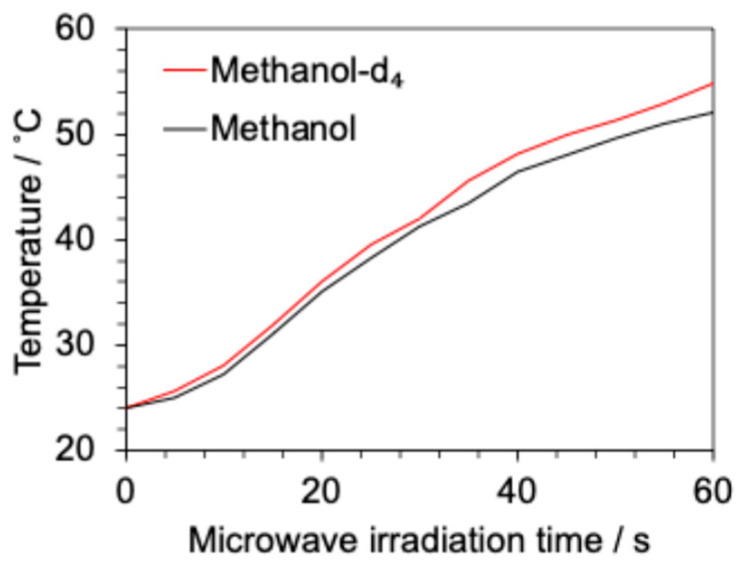
Temperature changes in methanol and methanol-*d*_4_ under microwave irradiation.

The dielectric factor results were verified by a heating experiment. Individually, 5 mL of methanol and methanol-*d*_4_ were irradiated, and the temperature change due to microwave heating (50 W) was monitored (Figure 4). This showed that methanol had a slightly higher heating efficiency than methanol-*d*_4_, which was consistent with the dielectric factor measurements. However, the difference in heating rate was small and is not likely the main cause of the high synthesis efficiency of heavy methylation by microwave irradiation.

We hypothesized that the improved efficiency of deuterated methylation in *N*-methylation reactions stems from differences in the interaction between methanol or methanol-*d*_4_ and the Co(acac)_2_ catalyst under microwave irradiation. Specifically, we hypothesized that microwave-induced cluster fragmentation is more dominant in deuterated methylation, resulting in a more pronounced facilitating effect. Therefore, we tested this hypothesis using molecular dynamics simulations.

A model with 1000 molecules of methanol and methanol-*d*_4_ were arranged within a 4 nm cubic box. To examine structural changes during heating, we tracked the time evolution of solvent clusters in methanol and methanol-*d*_4_ under microwave irradiation using MD trajectories (Figure S7). Microwave irradiation caused large clusters to fragment in both solvents, with a more pronounced effect in methanol-*d*_4_. Thus, deuterated molecules promote more extensive cluster breakdown due to their slower rotational relaxation and reduced O–D···O hydrogen-bond reorganization rate. The resulting phase shift in molecular orientation creates local structural inconsistencies that accelerate hydrogen-bond cleavage. These features also influence the solvation structure around Co(acac)_2_. Because the O–D···O network collapses more readily, the methanol-*d*_4_ solvation shell is more easily disrupted, improving access of substrates to catalytically active species and enhancing trideuteromethylation efficiency under microwave irradiation. Note that these simulation results should be considered as trend indicators, and further DFT calculations that explicitly incorporate microwave field effects are likely to be necessary for a more detailed understanding.

**Figure 5:**
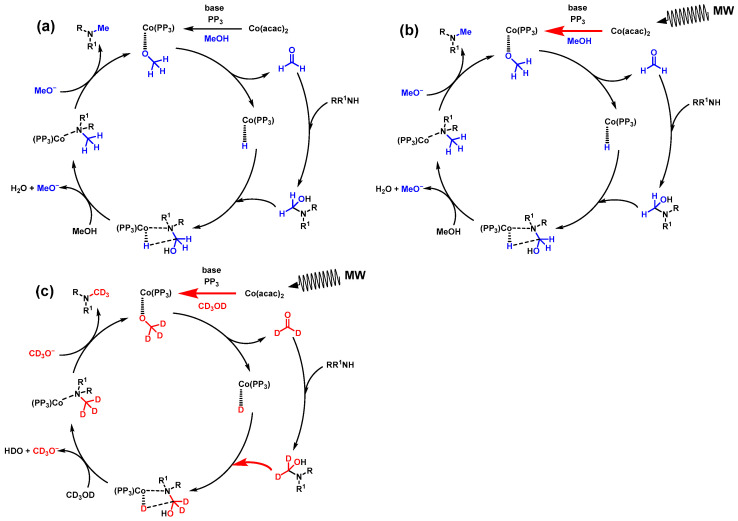
Models of the catalytic cycle (**a**) under CH conditions with methanol, (**b**) under MWH conditions with methanol, and (**c**) under MWH conditions with methanol-*d*_4_. Blue denotes non-labeled methanol and methyl groups, while red represents deuterium-labeled methanol and methyl groups. Red arrows indicate reaction steps expected to be accelerated by MW.

The catalytic cycles for the *N*-methylation reactions in this study are summarized in Figure 5. First, in the catalytic cycle under normal heating (Figure 5a), the acac ligand is removed from Co(acac)_2_, and a phosphorus ligand and a methoxide ion coordinate to Co, generating a Co active species [10]. This active species undergoes β-hydrogen elimination to form Co(PP_3_)-hydride and releases formaldehyde. The resulting formaldehyde reacts with an amine compound to generate an amino alcohol, which then reacts with Co(PP_3_)-hydride to generate a catalytic intermediate. Further hydrogen transfer occurs to form the Co(PP_3_)-methylamine complex, which then coordinates with a methoxide ion, regenerating the Co active species and releasing methylamine. While this proposed pathway is based on previous reports [10], alternative mechanisms, such as the formation of an imine intermediate via the dehydration of the amino alcohol followed by its hydrogenation at the cobalt center, cannot be ruled out. Given the presence of a base and the high-energy environment provided by microwave irradiation, such an imine-mediated process is also a plausible route.

The UV–vis absorption spectra shown in Figure 2 reveal that the consumption rate of Co(acac)_2_ is approximately four times faster under MWH conditions. This suggests that the conversion rate from Co(acac)_2_ to Co active species is accelerated by MWH as shown in Figure 5b, which is likely the main reason for the microwave-assisted reaction acceleration in the *N*-methylation reaction.

In the methanol-*d*_4_ system, molecular dynamics simulations suggest that the contact efficiency between the substrate and catalyst is improved. This is likely to increase the reaction rate of coordination of amino alcohols to Co(PP_3_)-hydride (Figure 5c). Furthermore, the isotope effect of deuterium substitution extends the lifetime of catalytic intermediates such as Co(PP_3_)-hydride, reducing ligand dissociation and suppressing side reactions, presumably contributing to improved yields. Thus, the remarkable effect of MWH in the methanol-*d*_4_ system is likely the result of the synergistic effects of three factors: (i) an increase in the rate of Co active species generation, (ii) an extension of the contact time between the amino alcohol and Co(PP_3_)-hydride, and (iii) suppression of side reactions due to isotope effects.

In addition, although speculative, it is possible that microwave heating suppressed the production of carbon monoxide as a side reaction. It is known that excessive oxidation of methanol by a metal catalyst produces formic acid, which then decomposes into hydrogen, water, and carbon monoxide [51]. The produced carbon monoxide may act as a catalyst poison, deactivating intermediates and potentially terminating the catalytic cycle. It is also suggested that the selective heating of the transition metal catalyst by microwaves may further enhance the rate of catalytically active species generation.

## 3. Materials and Methods

### 3.1. Microwave System

**Figure 6:**
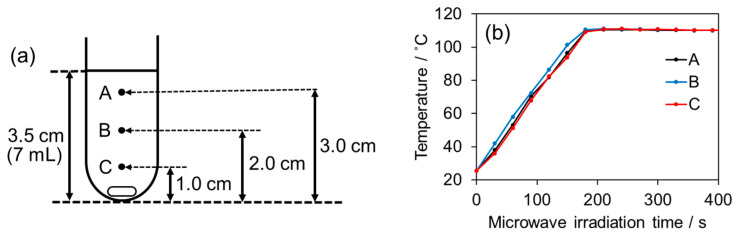
(**a**) Temperature measurement positions (A, B, C) when measuring the temperature of a reaction solution placed in a reaction vessel, and (**b**) the temperature change in the reaction temperature at those temperature measurement positions.

The MW apparatus used in this study was the µReactor Ex (Shikoku Keisoku Co., Ltd., Tadotsu, Japan), a microwave organic synthesis apparatus with a magnetic stirrer and controllable MW output, and a pressure-resistant tube (Ace Glass Co., Ltd., Vineland, NJ, USA) was used as the reaction vessel. Because microwave heating typically results in temperature variations depending on the location in the solution, this was confirmed in a preliminary experiment. Temperature uniformity was measured with a fiber optic thermometer under stirring conditions (500 rpm) using 7.0 mL of reaction solution with the MW output set to 10 W continuously. Measurement locations are points of A, B, and C in Figure 6a. As a result, the heating rates and final temperatures at all three points were within the error range, confirming the absence of heating non-uniformity (Figure 6b).

### 3.2. Chemical Reagents and Analytical Setup

All chemicals were purchased from Sigma-Aldrich Co. LLC (St. Louis, MO, USA), Thermo Fisher Scientific Inc. (Waltham, MA, USA), Tokyo Chemical Industry Co., Ltd. (TCI, Tokyo, Japan), Kanto Chemical Co., Inc. (Tokyo, Japan) or FUJIFILM Wako Pure Chemical Corporation (Osaka, Japan) and were used as received unless stated otherwise. Co(acac)_2_ was procured from TCI (product number B2681). NMR spectra were obtained at 25 °C on a JEOL JMTC-500 spectrometer (500 MHz; JEOL Ltd., Akishima, Japan) using CDCl_3_ or DMSO-*d*_6_ as solvent. Data were processed using Delta software (version 6.3.0, JEOL Ltd., Japan). Chemical shifts were reported in parts per million (ppm) on the delta (*δ*) scale and were referenced to the residual solvent peaks (CDCl_3_: *δ* 7.26 ppm; DMSO-*d*_6_: *δ* 2.50 ppm). GC-MS analysis was performed on a GCMS-QP2010 system (Shimadzu Corporation, Nakagyo-ku, Japan) equipped with an Rtx-5 capillary column (30 m × 0.25 mm i.d., 0.25 μm film thickness). Detailed experimental procedures, product characterization, and GC quantification methods are provided in the SI.

## 4. Concluding Remarks

In this work, we established an efficient microwave-assisted, cobalt-catalyzed *N*-methylation platform using methanol as a benign C1 source, demonstrating its applicability to pharmaceutically relevant tertiary amines. Compared with conventional heating, microwave heating markedly improved product yields under milder conditions and shorter reaction times, and enabled product formation even for sterically demanding substrates. UV–vis analyses indicated that microwave irradiation accelerates the conversion of Co(acac)_2_ to the catalytically competent Co species, providing a key mechanistic basis for the observed rate enhancement. Importantly, the microwave effect was even more pronounced in *N*-trideuteromethylation with methanol-*d*_4_, affording imipramine-*d*_3_ hydrochloride in high yield. Molecular dynamics simulations suggested that the distinct microwave response of methanol-*d*_4_—characterized by slower dipole relaxation and enhanced cluster fragmentation—can increase catalyst–substrate contact, while kinetic isotope effects may stabilize catalytic intermediates and suppress side reactions. Collectively, these results position microwave-assisted cobalt catalysis as a practical, energy-efficient approach that improves energy efficiency, minimizes hazardous waste, and offers a scalable route to both methylated and deuterated amines relevant to pharmaceutical manufacturing.

## Figures and Tables

**Table 1:** Dielectric constant (*ε*′) and dielectric loss (*ε*″) of methanol and methanol-*d*_4_ at 25 °C or 60 °C, and the resulting tan *δ*.

	*ε*′	*ε*″	tan *δ* (=*ε*″/*ε*′)
Methanol (25 °C)	23.3	14.1	0.60
Methanol-*d*_4_ (25 °C)	21.4	14.3	0.67
Methanol (60 °C)	25.1	8.95	0.36
Methanol-*d*_4_ (60 °C)	24.1	9.34	0.39

## Data Availability

The original contributions presented in this study are included in the article/Supplementary Material. Further inquiries can be directed to the corresponding author.

## Supplementary Materials

The following supporting information can be downloaded at: https://www.mdpi.com/article/10.3390/molecules31071068/s1. References [10,13,52,53,54,55,56,57,58,59,60] are cited in the Supplementary materials.
