# Supplementary material for: Accelerated Cobalt-Catalyzed N-Methylation via Microwave-Induced Rapid Formation of Active Species Using Methanol and Methanol-d4
Source: Molecules. 2026 Mar 24;31(7):1068. doi: 10.3390/molecules31071068 (PMC13075069; doi:10.3390/molecules31071068)
Supplement: Supplementary file 1 [file molecules-31-01068-s001.zip › molecules-4166938-supplementary.pdf]

Microwave-Assisted Cobalt-Catalyzed *N*-Methylation and *N*-Trideuteromethylation Using Methanol and Methanol-d_4: Mechanistic Insights and Molecular Dynamics Analysis

Miki Takizawa [1], Takahiro Yamane [1], Akinobu Matsumoto [2], Takashi Miyazawa [2], Satoshi Horikoshi [1*]

[1]Sophia University, Department of Materials and Life Sciences, Faculty of Science and Technology, Sophia University, 7-1 Kioicho, Chiyoda-ku, Tokyo 102-8554, Japan
[2] Wisdom Pool Research Institute G.K., 32F Tokyo Midtown Yaesu Yaesu Central Tower, 2-1-1, Yaesu, Chuo-ku, Tokyo,104-0082 Japan

* Correspondence: horikosi@sophia.ac.jp (SH)

Supporting Information: Confirmation of each synthesis method and its yield

Chemical reagents and analytical setup

All chemicals were purchased from Sigma-Aldrich Co. LLC, Thermo Fisher Scientific Inc., Tokyo Chemical Industry Co., Ltd., Kanto Chemical Co., Inc. or FUJIFILM Wako Pure Chemical Corporation and were used as received unless stated otherwise. The specific origins and product numbers of the key reagents used in this study are summarized in Table S1. NMR spectra were obtained at 25 °C on a JEOL JMTC-500 spectrometer (500 MHz) using $CDCl_3$ or DMSO-d_6 as solvent. Chemical shifts were reported in parts per million (ppm) on the delta (δ) scale and were referenced to the residual solvent peaks ($CDCl_3$: δ 7.26 ppm; DMSO-d_6: δ 2.50 ppm). Splitting patterns were designated as fol-lows: s, singlet; d, doublet; t, triplet; q, quartet; quin, quintet; m, multiplet; and br, broad. For the catalytic synthesis of 6-dimethylamino-1-hexanol, the yield was determined by GC analysis using the internal standard method with decane (TCI, >99.0%, product number D0011) as an internal standard. The identity of the product was confirmed by comparing its retention time with an authentic sample of 6-dimethylamino-1-hexanol purchased from TCI (product number D1664, >97.0%). A calibration curve was established using the authentic sample and the internal standard to ensure accurate quantification. GC-MS analysis was performed on a GCMS-QP2010 system (Shimadzu Corporation) equipped with an Rtx-5 capillary column (30 m × 0.25 mm i.d., 0.25 μm film thickness). Helium was used as the carrier gas.

Table S1. List of materials and reagents with their suppliers and product numbers. TCI: Tokyo Chemical Industry Co., Ltd.; SA: Sigma-Aldrich Co. LLC; FWP: Fujifilm Wako pure Co.; KC: Kanto Chemical Co. Ltd.; TFS: Thermo Fisher Scientific Inc.

Reagent	Supplier	Product No.
6-Amino-1-hexanol	TCI	A1027
Potassium *tert*-butoxide (KO*t*Bu)	TCI	P1008
Potassium Phosphate (K_3PO_4)	TCI	T3998
Tris[2-(diphenylphosphino)ethyl]phosphine (PP_3)	SA	327697-1G
Cobalt(II) acetylacetonate ($Co(acac)_2$)	TCI	B2681
4-*tert*-Butylbenzylamine	TCI	B1587
1-Naphthaldehyde	TCI	N0002
10,11-Dihydro-5*H*-dibenz[*b*,*f*]azepine	TCI	D0269
1,3-Dibromopropane	TCI	D0202
40%Methylamine Methanol Solution	FWP	137-10392
Methanol	FWP	131-01826
Methanol-d_4, 99.8 atom % D	TFS	321280250
Sodium Borohydride ($NaBH_4$)	TCI	S0480
Boric Acid (H_3BO_3)	FWP	026-15715
Acetic Acid (AcOH)	FWP	017-00256
Hydrochloric Acid (HCl)	FWP	087-01076
Lithium amide ($LiNH_2$)	SA	213217-5G
Toluene	FWP	201-01876
Sodium Hydroxide (NaOH)	FWP	192-15985
Chloroform-d_1, 99.8 atom % D	KC	07660-97
Dimethyl Sulfoxide-*d*6 99.9atom % D	TCI	D0381

Synthesis of 6-dimethylamino-1-heanol.

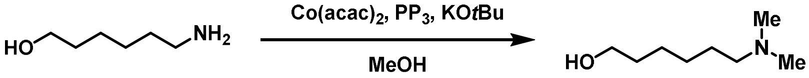

Scheme S1. Synthesis of 6-dimethylamino-1-hexanol

In a 15 mL pressure-resistant vessel (Ace Glass, Inc.) equipped with a thermowell, add a stir bar, 6-amino-1-hexanol (200 mg, 1.7 mmol), potassium tert-butoxide (KO*t*Bu, 19 mg, 0.17 mmol), Tris[2-(diphenylphosphino)ethyl]phosphine (PP_3, 228 mg, 0.34 mmol), and methanol (5 mL) were added. After bubbling the reaction solution with nitrogen for 5 min, cobalt acetylacetonate ($Co(acac)_2$, 87 mg, 0.34 mmol) was added. The mixture was reacted under heating either by a conventional heating (EYELA: RCH-1000) or microwave heating (Shikoku Instrumentation Co., Ltd.: μReactor Ex) at 100 °C. For the microwave heating settings, Peak was set to 50%, Duty to 100%, and the cycle to 10 s (P: 10%, I: 100 s, D: 6 s). The reaction time was set to 4 hrs for both heating methods. After the reaction, decane (internal standard) was added directly to the crude reaction mixture. The solution was then extracted with ethyl acetate and washed with water. The organic layer was dehydrated with sodium sulfate, filtered through cotton plugs, and the resulting filtrate was analyzed by GC-MS. The GC yield was determined based on the internal standard added prior to the workup. The synthesis was performed according to a modified procedure from literature [10].

Synthesis of butenafine precursor.

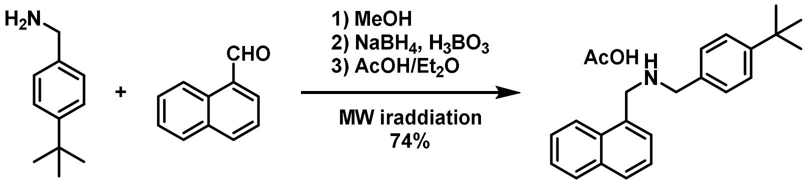

Scheme S2. Synthesis of butenafine precursor.

A stir bar, 4-*tert*-butylbenzylamine (1.9 g, 12.2 mmol), 1-naphthaldehyde (1.9 g, 12.2 mmol), and methanol (80 mL) were added to a three-neck flask. A reflux condenser was attached to the reaction vessel, and a balloon filled with nitrogen gas was connected. The reaction was conducted at 64 °C using a microwave oscillator (Shikoku Instrumentation Co., Ltd.: μ Reactor Ex). The heating settings were Peak 20%, Duty 100%, and a cycle time of 10 s (P: 10%, I: 100 s, D: 6 s) for 30 min. The formation of the Schiff base of the imine was confirmed by GC-MS. Boric acid (1.1 g, 18.3 mmol) and sodium borohydride (690 mg, 18.3 mmol) were slowly added to the reaction solution and stirred for 10 min. Subsequently, the reaction was conducted at 65 °C using a microwave oscillator (Shikoku Instrumentation Co., Ltd.: μ Reactor Ex). The heating settings were Peak 15%, Duty 100%, and a cycle time of 10 s (P: 10%, I: 100 s, D: 6 s), with heating for 1 hour. After heating, the disappearance of the Schiff base was confirmed by thin-layer chromatography (TLC). To quench the reaction, potassium carbonate aqueous solution was added to the reaction mixture, adjusting it to neutral to weakly basic conditions. The mixture was then extracted with dichloromethane and washed with water. Sodium sulfate was added to the organic layer to dehydrate it. The solution was transferred to a round-bottom flask via cotton-stoppered filtration and concentrated using an evaporator. After vacuum drying, the residue was dissolved in diethyl ether (5 mL). A white precipitate formed upon blowing acetic acid gas over the solution. The precipitate was filtered under suction and washed with a small amount of diethyl ether and heptane to obtain the white solid butenafine precursor. Vacuum drying yielded the desired product (3.3 g: 9.0 mmol, 75%). The synthesis was performed according to a modified procedure from literature [52]. It should be noted that our ^{1}H NMR spectrum was recorded for the acetate salt in DMSO-d_6, whereas the literature [52] reports the data for the free base in $CDCl_3$. The formation of the acetate salt was confirmed by the characteristic singlet at δ 1.90 ppm (3H, CH_3COO^-). ^{1}H NMR (500 MHz, DMSO-d_6) δ 8.15–8.12 (m, 1H), 7.92–7.89 (m, 1H), 7.81 (d, J = 8.0 Hz, 1H), 7.54–7.44 (m, 4H), 7.35–7.29 (m, 4H), 4.13 (s, 2H), 3.76 (s, 2H), 1.90 (s, 3H), 1.27 (s, 9H) (Figure S1).

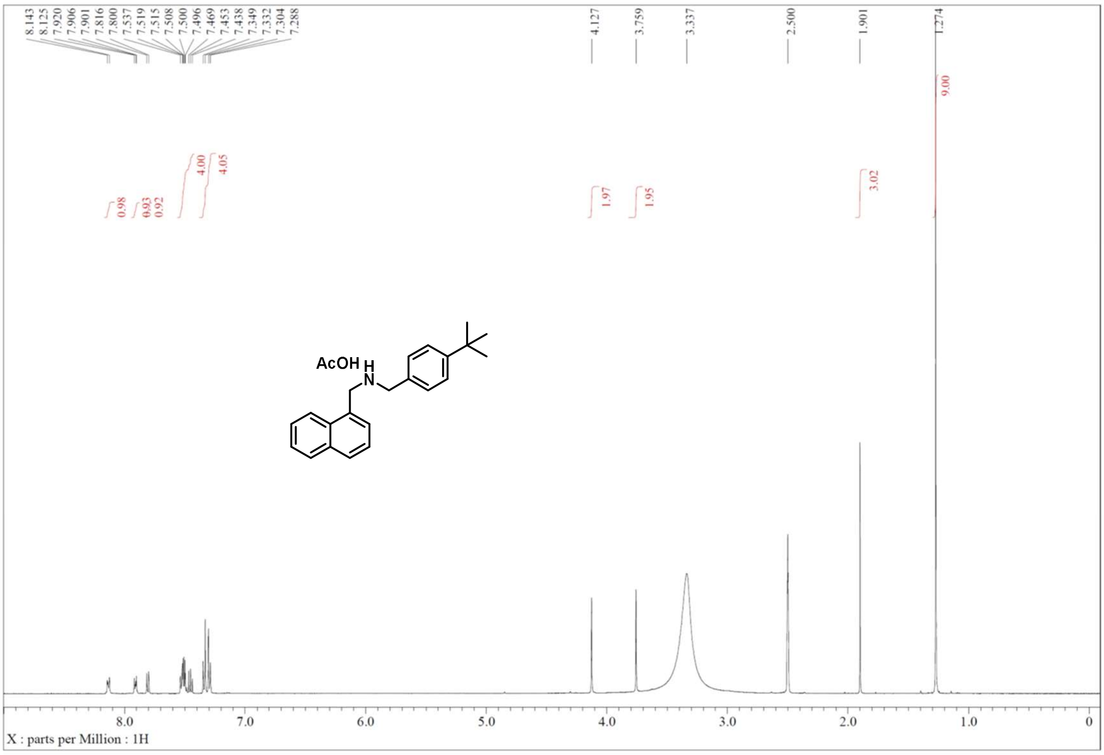

Figure S1. ^{1}H NMR (500 MHz, DMSO-d_6) of butenafine precursor.

Synthesis of butenafine hydrochloride.

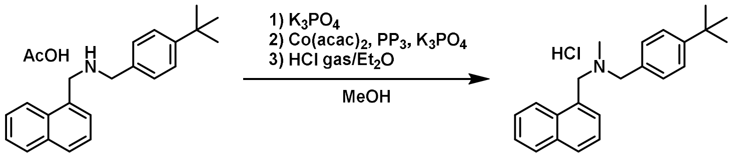

Scheme S3. Synthesis of butenafine hydrochloride.

In a 15 mL pressure-resistant vessel (Ace Glass, Inc.) equipped with a thermowell, a stir bar, butenafine precursor (363 mg, 1.0 mmol), and methanol (6 mL) were added. After nitrogen bubbling for 10 min, tripotassium phosphate hydrate (212 mg, 1.0 mmol) was added and stirred at room temperature. Subsequently, $Co(acac)_2$ (103 mg, 0.4 mmol), PP_3 (260 mg, 0.4 mmol), and tripotassium phosphate hydrate (640 mg, 3 mmol) were added to the reaction solution. After 10 min of nitrogen bubbling, the mixture was heated to 140 °C using either conventional heating (EYELA: RCH-1000) or microwave heating (Shikoku Instrumentation Co., Ltd.: µReactor Ex). For the microwave oscillator heating settings, Peak was set to 40%, Duty to 100%, and the cycle to 10 s to proceed the reaction (P: 10%, I: 100 s, D: 6 s). The reaction time was set to 8.5 hrs. After heating, a 2 cm layer of Celite and a 5 cm layer of silica gel were packed into a Kiriyama funnel, and suction filtration was performed using ethyl acetate as the developing solvent. TLC confirmed the disappearance of the butenafine precursor component. The filtrate was concentrated using an evaporator to yield a deep yellow oil. This was purified by column chromatography (elution solvent: ethyl acetate/hexane = 1/20). After concentration, the obtained oil was dissolved in diethyl ether. Hydrogen chloride gas was bubbled through the solution, confirming the precipitation of a white precipitate. Vacuum filtration was performed, followed by washing with diethyl ether. The resulting white solid was vacuum dried to afford butenafine hydrochloride (18 mg, 0.05 mmol, 5% yield). In contrast, when conventional heating was employed, the formation of the product was confirmed only in trace amounts by GC-MS analysis, and the product could not be isolated by column chromatography. NMR spectroscopy of the white solid confirmed its identity as butenafine hydrochloride. The synthesis was performed according to a modified procedure from literature [10]. It should be noted that our ^{1}H NMR spectrum was recorded for the hydrochloride salt in DMSO-d_6, whereas the literature [52] reports the data for the free base in $CDCl_3$. The formation of the hydrochloride salt was confirmed by the characteristic singlet at δ 10.04 ppm (1H, NH^+). ^{1}H NMR (500 MHz, DMSO-d_6) δ 10.04 (br s, 1H), 8.07–8.00 (m, 3H), 7.84–7.83 (m, 1H), 7.62–7.51 (m, 7H), 4.92–4.89 (m, 1H), 4.68–4.64 (m, 1H), 4.52–4.41 (m, 2H), 2.61 (s, 3H), 1.31 (s, 9H) (Figure S2).

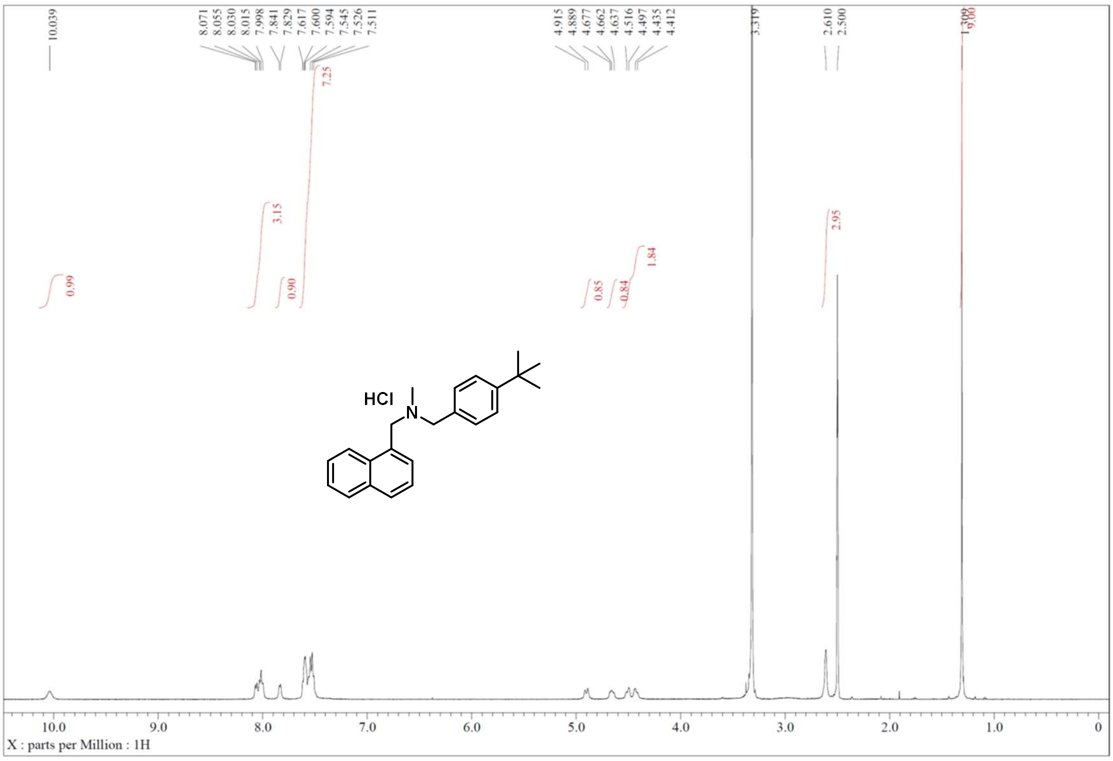

Figure S2. ^{1}H NMR (500 MHz, DMSO-d_6) of butenafine hydrochloride.

Synthesis of desipramine precursor.

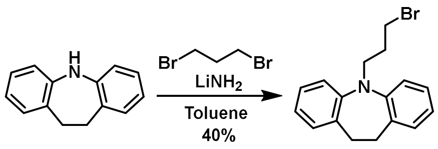

Scheme S4. Synthesis of desipramine precursor.

In a 15 mL pressure-resistant vessel (Ace Glass, Inc.) equipped with a thermowell, a stir bar, dihydrodibenzoazepine (976 mg, 5.0 mmol), 1,3-dibromopropane (1.5 g, 7.5 mmol), and toluene (5 mL) were added. After bubbling nitrogen for 10 min, lithium amide (138 mg, 6.0 mmol) was added. The reaction was conducted at 125 °C using a microwave reactor (Shikoku Instrumentation Co., Ltd.: μReactor Ex). The microwave heating settings were Peak 100%, Duty 100%, and a cycle time of 5 s (P: 10%, I: 100 s, D: 6 s). The reaction time was set to 8.5 hrs. After heating, the reaction solution was quenched by adding 5 mL of deionized water. The mixture was then extracted with ethyl acetate and washed with water. The resulting organic layer was dehydrated with sodium sulfate, and the solvent was removed using an evaporator. The obtained oily mixture was purified by column chromatography (elution solvent: hexane). After concentration in the evaporator, the resulting oil was subjected to NMR spectroscopy, confirming it as the target alkyl bromide (632 mg: 2.0 mmol, 40%). The synthesis was adapted from the procedure reported in patent [53] with a minor modification of the alkylating agent (using the dibromo derivative instead of the bromo-chloro analogue). It should be noted that the ^{1}H NMR spectrum was consistent with the patent [53], except for the chemical shift of the terminal methylene protons. A triplet observed at δ 3.43 ppm was assigned to the –CH_2Br group, appearing at a higher field compared to the –CH_2Cl group (δ 3.57 ppm) reported in the patent [53] due to the lower electronegativity of bromine relative to chlorine. ^{1}H NMR (500 MHz, $CDCl_3$) δ 7.15–7.07 (m, 6H), 6.95–6.92 (m, 2H), 3.90 (t, J = 6.6 Hz, 2H), 3.43 (t, J = 6.6 Hz, 2H), 3.16 (s, 4H), 2.13 (quin, J = 6.5 Hz, 2H) (Figure S3).

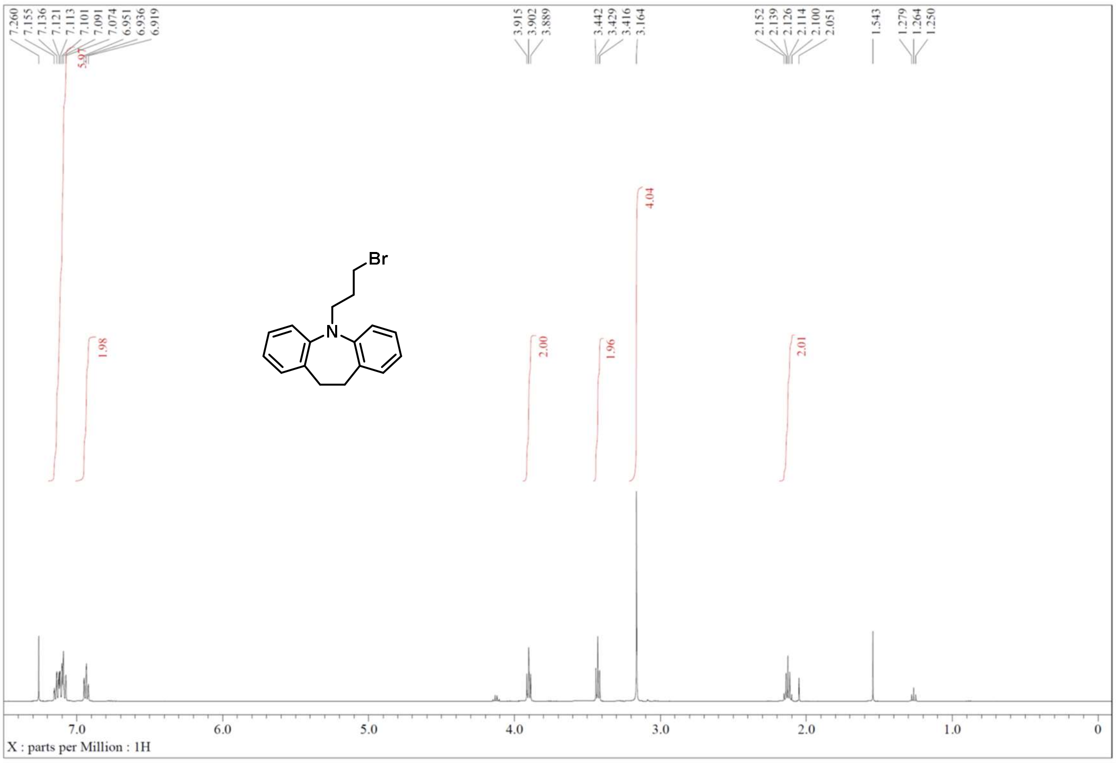

Figure S3. ^{1}H NMR (500 MHz, $CDCl_3$) of desipramine precursor.

Synthesis of desipramine hydrochloride.

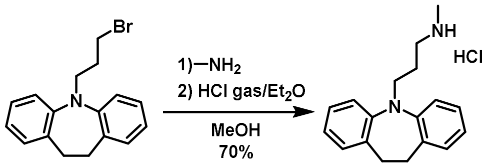

Scheme S5. Synthesis of desipramine hydrochloride.

The synthesis of desipramine hydrochloride was carried out following standard S_N2 amination procedures. To a 50 mL conical flask equipped with a stir bar, the alkyl bromide precursor of imipramine (1.59 g, 5.0 mmol), diethyl ether (1 mL), potassium carbonate (695 mg, 5.0 mmol), and a 40% methylamine solution in methanol (6 mL) were added. Connect a reflux condenser to a balloon filled with nitrogen gas and stir. (695 mg, 5.0 mmol), and 40% methylamine methanol solution (6 mL) to a 50 mL conical flask. A reflux condenser and a balloon filled with nitrogen gas were attached, and stirring was performed at room temperature. After 2 hrs, the reaction mixture was analyzed by GC-MS, confirming that the starting materials were consumed and the target molecule, desipramine, was synthesized. After 16 hrs, the mixture was extracted with ethyl acetate and washed with water. The organic layer was dried over anhydrous sodium sulfate, filtered, and concentrated under reduced pressure to yield a crude oil. The resulting oil was dissolved in diethyl ether (10 mL), and hydrogen chloride gas was bubbled through for 5 min to confirm the precipitation of a white solid. The white solid was obtained by suction filtration while washing with diethyl ether. Vacuum drying was performed, and NMR spectroscopy of the white solid confirmed it as desipramine hydrochloride (1.06 g: 3.5 mmol, 70%). The synthesis of desipramine hydrochloride was confirmed by ^1H NMR spectroscopy. The obtained chemical shifts were consistent with the literature values for desipramine free base [53], with characteristic downfield shifts observed for the *N*-methyl (δ 2.47 ppm) and *N*-methylene (δ 2.88 ppm) protons due to the formation of the hydrochloride salt. Furthermore, the appearance of a broad singlet at δ 8.26 ppm (2H), assigned to the ammonium protons, unequivocally supported the successful preparation of the salt form. ^1H NMR (500 MHz, DMSO-d_6) δ 8.26 (br s, 2H), 7.16–7.12 (m, 6H), 6.95–6.92 (m, 2H), 3.77 (t, J = 6.6 Hz, 2H), 3.09 (s, 4H), 2.88 (t, J = 7.4 Hz, 2H), 2.47 (s, 3H), 1.78 (quin, J = 7.0 Hz, 2H) (Figure S4).

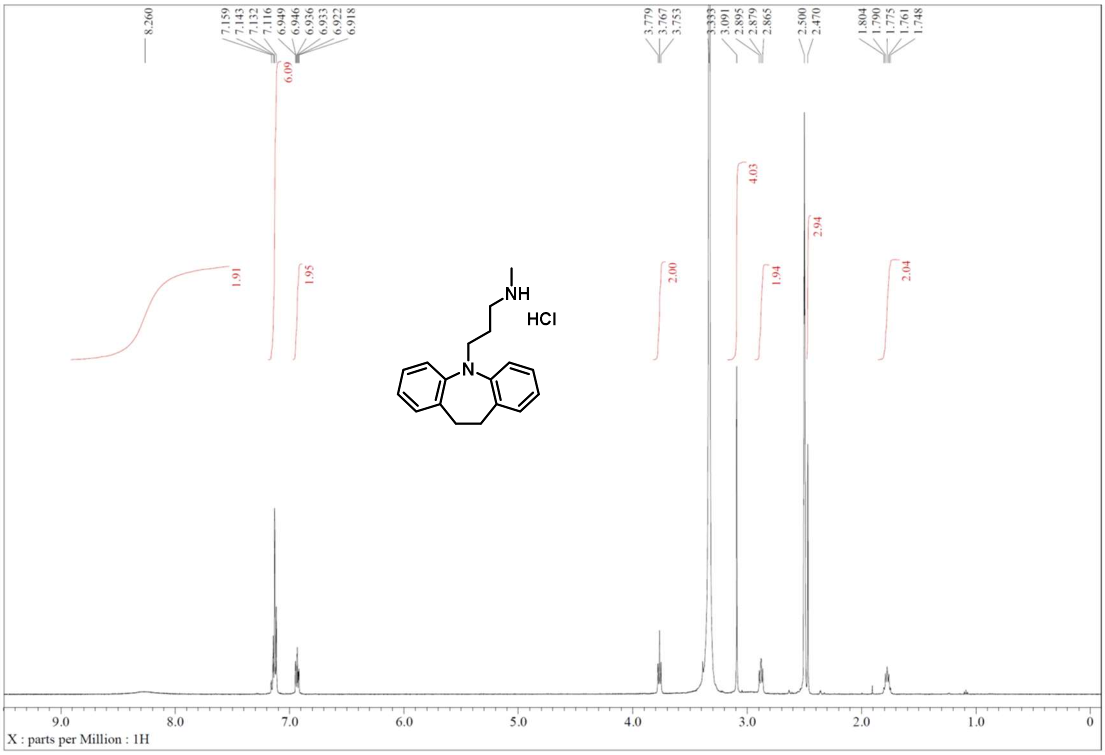

Figure S4. ^{1}H NMR (500 MHz, DMSO-d_6) of desipramine hydrochloride.

Synthesis of imipramine hydrochloride.

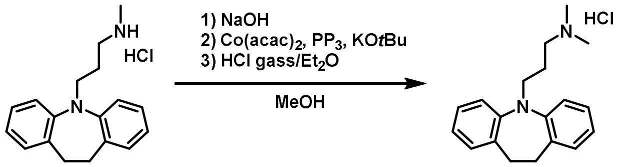

Scheme S6. Synthesis of imipramine hydrochloride.

Add desipramine hydrochloride (180 mg, 0.59 mmol), NaOH (120 mg), H_2O (1 mL), and methanol (5 mL) to a 1-neck flask and react at room temperature. After 3 hrs, extract the reaction solution with ethyl acetate and wash with water. The resulting organic layer was dehydrated with sodium sulfate, and the solvent was removed using an evaporator. The pale-yellow oil obtained was dissolved in methanol (4 mL) and added to a 15 mL pressure-resistant vessel (Ace Glass, Inc) equipped with a thermowell. PP_3 (80 mg, 0.12 mmol), and potassium tert-butoxide (KO*t*Bu, 66 mg, 0.59 mmol) were added to the reaction solution and bubbled with nitrogen for 5 min. $Co(acac)_2$ (31 mg, 0.12 mmol) was added to the reaction solution, and the mixture was reached at 100 °C using either conventional heating (EYELA: RCH-1000) or microwave heating (Shikoku Instrumentation Co., Ltd.: μReactor Ex). For the microwave oscillator heating settings, Peak was set to 10%, Duty to 100%, and the cycle to 10 s to proceed the reaction (P: 10%, I: 100 s, D: 6 s). The reaction time was set to 1 hr. After heating, the reaction solution was analyzed by GC-MS to calculate the yield of the starting material and the target product.

The reaction solution was quenched with deionized water, extracted with ethyl acetate, and washed with water. The resulting organic layer was dehydrated with sodium sulfate, and the solvent was removed using an evaporator. The dark brown oily mixture obtained was purified using activated alumina (elution solvent: ethyl acetate/hexane = 2/1 + 1% triethylamine). After concentration, the pale-yellow oil obtained was identified as imipramine by NMR measurement.

The purified imipramine was dissolved in diethyl ether (2 mL), and the addition of hydrochloric acid gas confirmed the precipitation of a white solid. Vacuum filtration was performed while washing with diethyl ether, followed by vacuum drying to afford imipramine hydrochloride as a white solid. Under microwave heating, the product was obtained in 79% yield (148 mg, 0.47 mmol). In contrast, conventional heating afforded the product in 42% yield (79 mg, 0.25 mmol). NMR spectroscopy of the white solid confirmed its identity as imipramine hydrochloride. The synthesis was performed according to a modified procedure from literature [10]. The ^{1}H NMR data of the obtained imipramine hydrochloride in DMSO-d_6 were consistent with the reported values for the

free base in $CDCl_3$ [13], taking into account the solvent effect and salt formation. Specifically, the appearance of a broad singlet at δ 10.08 ppm (1H) assigned to the ammonium proton (NH^+) and the characteristic shifts of the *N*,*N*-dimethyl and adjacent methylene signals confirmed the successful conversion to the hydrochloride salt. ^{1}H NMR (500 MHz, DMSO-d_6) δ 10.08 (br s, 1H), 7.16–7.12 (m, 6H), 6.96–6.91 (m, 2H), 3.76 (t, J = 6.9 Hz, 2H), 3.09 (s, 4H), 3.05 (t, J = 7.7 Hz, 2H), 2.64 (s, 6H), 1.84 (quin, J = 7.3 Hz, 2H) (Figure S5).

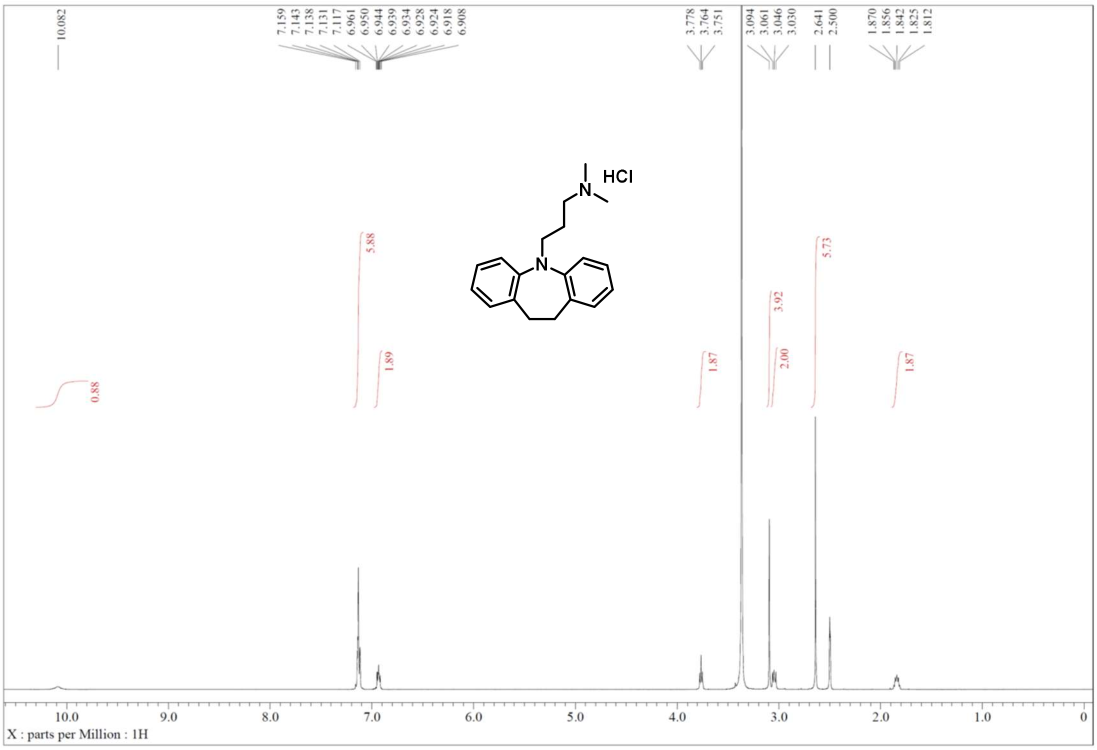

Figure S5. ^{1}H NMR (500 MHz, DMSO-d_6) of Imipramine hydrochloride.

Synthesis of imipramine-*d*3 hydrochloride.

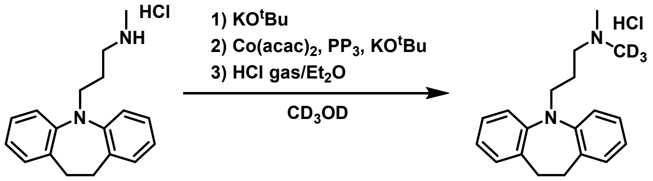

Scheme S7. Synthesis of imipramine-*d*3 hydrochloride.

Add desipramine hydrochloride (200 mg, 0.66 mmol), KO*t*Bu (74 mg, 0.66 mmol), and methanol-*d*4 (4 mL) to a 15 mL pressure-resistant vessel (Ace Glass, Inc.) and react at room temperature. After 1 hr, PP_3 (87 mg, 0.13 mmol) and KO*t*Bu (74 mg, 0.66 mmol) were added to the reaction solution and nitrogen was bubbled through for 5 min. $Co(acac)_2$ (33 mg, 0.13 mmol) was added to the reaction solution and the mixture was reached at 100 °C using either conventional heating (EYELA: RCH-1000) or microwave heating (Shikoku Instrumentation Co., Ltd.: µReactor Ex). For the microwave oscillator heating settings, Peak was set to 10%, Duty to 100%, and the cycle to 10 s to proceed the reaction (P: 10%, I: 100 s, D: 6 s). The reaction time was set to 6 hrs. After heating, the reaction solution was analyzed by GC-MS to calculate the yield of the starting material and the target product.

The reaction solution was quenched with deionized water, extracted with ethyl acetate, and washed with water. The resulting organic layer was dehydrated with sodium sulfate, and the solvent was removed using an evaporator. The dark brown oily mixture obtained was purified using activated alumina (elution solvent: ethyl acetate/hexane = 2/1 + 1% triethylamine). After concentration, the pale-yellow oil obtained was identified as imipramine-*d*3 by NMR measurement.

The purified imipramine-*d*3 was dissolved in diethyl ether (2 mL), and the addition of hydrochloric acid gas confirmed the precipitation of a white solid. Vacuum filtration was performed while washing with diethyl ether, yielding a white solid. Vacuum drying was conducted to afford imipramine-*d*3 hydrochloride. Under microwave heating, the product was obtained in 95% yield (201 mg, 0.63 mmol), while conventional heating resulted in a significantly lower yield of 32% (68 mg, 0.21 mmol). NMR spectroscopy of the white solid confirmed its identity as imipramine-*d*3 hydrochloride. The synthesis was performed according to a modified procedure from literature [10]. The ^{1}H NMR spectrum of imipramine-*d*3 hydrochloride was consistent with that of the non-labeled imipramine hydrochloride, with the exception of the *N*-methyl signal. The integral value of the signal at δ 2.65–2.64 ppm decreased to 3H (compared to 6H in the non-labeled form), confirming the successful incorporation of one CD_3 group. The formation of the

hydrochloride salt was supported by the characteristic ammonium proton observed at δ 10.13 ppm. ^{1}H NMR (500 MHz, DMSO-d_6) δ 10.13 (br s, 1H), 7.16–7.09 (m, 6H), 6.98–6.91 (m, 2H), 3.76 (t, J = 6.6 Hz, 2H), 3.10 (s, 4H), 3.05–3.03 (m, 2H), 2.65–2.64 (m, 3H), 1.84 (quin, J = 7.3 Hz, 2H) (Figure S6).

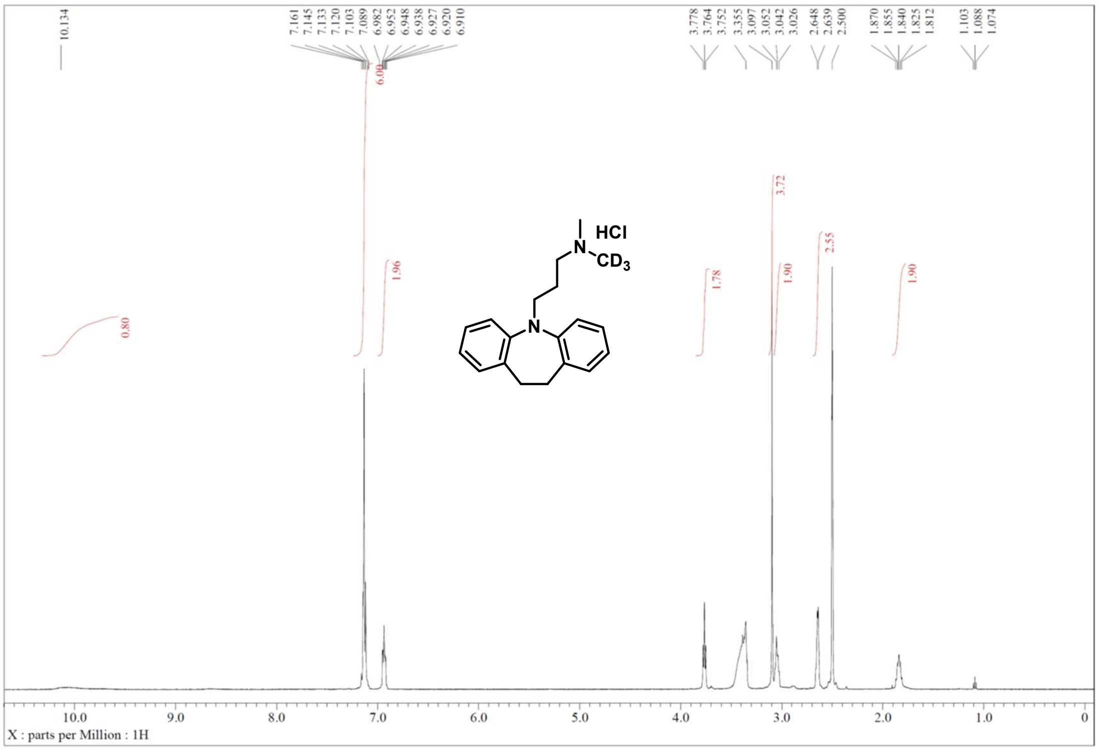

Figure S6. ^{1}H NMR (500 MHz, DMSO-d_6) of imipramine-d_3 hydrochloride.

Molecular dynamics (MD) simulations under microwave irradiation

It is important to note the scope and limitations of the present computational approach. Classical MD simulations employing the OPLS-AA force field cannot describe quantum mechanical phenomena such as zero-point energy (ZPE) or bond cleavage events, which govern the chemical kinetic isotope effect (KIE) of the rate-limiting step (e.g., β-hydrogen elimination). Therefore, the purpose of this MD study is strictly limited to elucidating the macroscopic physical phenomena of microwave dielectric heating and the resulting bulk solvent dynamics. The chemical reaction mechanisms are beyond the scope of this classical model. It used the molecular dynamics simulation software GROMACS to investigate the dynamic behavior of an ensemble of methanol and methanol-d_4 molecules under a 2.45 GHz electric field. In this calculation, the charge and

Lennard-Jones parameters were set identically for both systems, and the following results are purely attributable to the kinetic isotope effect associated with increasing atomic mass. Using GROMACS software version 2025.2, it constructed a model with 1000 molecules of methanol and methanol-d_4 arranged within a 4 nm cubic box (Figure S7). The MD simulations used the OPLS-AA force field [54], which was developed for liquid simulations [55]. The structure of the system was calculated by the steepest descent method [56], with a load of 1000 kJ mol^{-1} nm^{-1} and a maximum step distance of 0.01 nm. The system was then thermodynamically balanced using the leapfrog algorithm in the NVT and NPT ensembles. The simulation time was 200 ps, with a V-rescale of 300 K and a C-rescale of 1 bar, where the temperature and pressure were kept constant. Finally, the MD generation step utilized the velocity Werle method, which determines the kinetic energy using the half-step averaged velocity of the NVE ensemble, with the volume and energy kept constant from the initial structure of the previous step. The MD generation step was then performed for 100 ns with a continuous electric field of 0.5 V nm^{-1} at 2.45 GHz in the x-axis direction only [57]. The molecular dynamics simulation trajectory was analyzed using GROMACS, and the Visual Molecular Dynamics (VMD) 1.9.4a53 [58] package was used for visualization and detailed analysis. Based on the obtained simulation trajectories, the dipole moment autocorrelation function [59] and the time evolution of the temperature of the NVE ensemble [60] were analyzed to evaluate the differences in the dynamic behavior and response to the external electric field between methanol and methanol-d_4. Additionally, cluster size analysis was performed using a distance criterion of 0.35 nm between oxygen atoms to evaluate the fragmentation of the hydrogen-bonded networks.

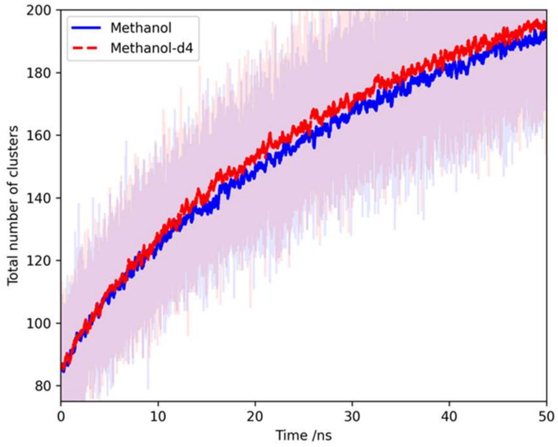

Figure S7. Computational results showing the change in the number of methanol molecular clusters over time when a microwave electric field (2.45 GHz) is applied. The

clusters were determined from MD simulation trajectories using an O–O distance cutoff of 0.35 nm.

Reference

The reference numbers match those in the paper.

10. Liu, Z.; Yang, Z.; Yu, X.; Zhang, H.; Yu, B.; Zhao, Y.; Liu, Z. Efficient Cobalt-Catalyzed Methylation of Amines Using Methanol. Adv. Synth. Catal. 2017, 359, 4278−4283; https://doi.org/10.1002/adsc.201701044
13. Sarki, N.; Goyal, V.; Tyagi, N.K.; Puttaswamy; Narani, A.; Ray, A.; Natte, K. Simple $RuCl_3$-catalyzed *N*-Methylation of Amines and Transfer Hydrogenation of Nitroarenes using Methanol. ChemCatChem 2021, 13, 1722−1729; https://doi.org/10.1002/cctc.202001937
52. Porras, A.M.G.; Terra, B.S.; Braga, T.C.; Magalhães, T.F.F.; Martins, C.V.B.; da Silva, D.L.; Baltazar, L.M.; Gouveia, L.F.; de Freitas, G.J.C.; Santos, D.A.; Resende-Stoianoff, M.A.; Fuchs, B.B.; Mylonakis, E.; de Freitas, R.P.; de Fátima, Â. Butenafine and analogues: An expeditious synthesis and cytotoxicity and antifungal activities, *J. Adv. Res.* **2018**, *14*, 81–91; https://doi.org/10.1016/j.jare.2018.06.004
53. Zhang, W.; Gu, X.; Allums, S.; Riggs-Sauthier, J. Oligomer-tricyclic conjugates as Analgesics, Oligomer-tricyclic conjugates as Analgesics, WO patent 2011091050, **2011**.
54. Jorgensen, W.L.; Maxwell, D.S.; Tirado-Rives, J. Development and Testing of the OPLS All-Atom Force Field on Conformational Energetics and Properties of Organic Liquids. *J. Am. Chem. Soc.* **1996**, *118*, 11225–11236; https://doi.org/10.1021/ja9621760
55. Doherty, B.; Acevedo, O. OPLS Force Field for Choline Chloride-Based Deep Eutectic Solvents. *J. Phys. Chem. B* **2018**, *122*, 9982–9993; https://doi.org/10.1021/acs.jpcb.8b06647
56. Arfken, G.B.; Weber, H.J.; Harris, F.E. *Mathematical Methods for Physicists: A Comprehensive Guide*, Seventh Edition.; Academic Press: Waltham, MA, USA, **2013**, ISBN 978-0-12-384654-9.
57. Berendsen, H.J.C.; van der Spoel, D.; van Drunen, R. GROMACS: A message-passing parallel molecular dynamics implementation. *Comput. Phys. Commun.* **1995**, *91*, 43–56; https://doi.org/10.1016/0010-4655(95)00042-E

58. Humphrey, W.; Dalke, A.; Schulten, K. VMD - Visual Molecular Dynamics. *J. Mol. Graph.* **1996**, *14*, 33–38; https://doi.org/10.1016/0263-7855(96)00018-5
59. Kubo, R. Statistical-Mechanical Theory of Irreversible Processes. I. General Theory and Simple Applications to Magnetic and Conduction Problems. *J. Phys. Soc. Jpn.* **1957**, *12*, 570–586; https://doi.org/10.1143/JPSJ.12.570
60. Frenkel, D.; Smit, B. Understanding Molecular Simulation: From Algorithms to Applications, 2nd ed.; Academic Press: San Diego, CA, USA, **2002**, ISBN 978-0-12-267351-1.
